# Nighttime eating and breast cancer among Chinese women in Hong Kong

**DOI:** 10.1186/s13058-017-0821-x

**Published:** 2017-03-17

**Authors:** Mengjie Li, Lap Ah Tse, Wing-cheong Chan, Chi-hei Kwok, Siu-lan Leung, Cherry Wu, Wai-cho Yu, Priscilla Ming-yi Lee, Koon-ho Tsang, Sze-hong Law, Roel Vermeulen, Fangyi Gu, Neil E. Caporaso, Ignatius Tak-sun Yu, Feng Wang, Xiaohong Rose Yang

**Affiliations:** 1 JC School of Public Health and Primary Care, the Chinese University of Hong Kong, Hong Kong SAR, China; 2 Department of Surgery, North District Hospital, Hong Kong SAR, China; 3 Department of Oncology, Princess Margaret Hospital, Hong Kong SAR, China; 4 Department of Surgery, Pamela Youde Nethersole Eastern Hospital, Hong Kong SAR, China; 5 Department of Pathology, North District Hospital, Hong Kong SAR, China; 6 Department of Medicine, Princess Margaret Hospital, Hong Kong SAR, China; 7 Department of Pathology, Yan Chai Hospital, Hong Kong SAR, China; 8 Department of Surgery, Yan Chai Hospital, Hong Kong SAR, China; 9 Institute for Risk Assessment Sciences, Utrecht University, Utrecht, Netherlands; 10 Genetic Epidemiology Branch, Division of Cancer Epidemiology & Genetics, National Cancer Institute, National Institutes of Health, Bethesda, USA

**Keywords:** Nighttime eating behavior, Dietary factors, Breast cancer

## Abstract

**Background:**

A novel line of research suggests that eating at nighttime may have several metabolic consequences that are highly relevant to breast cancer. We investigated the association between nighttime eating habits after 10 p.m. and breast cancer in Hong Kong women.

**Methods:**

A hospital-based case-control study was conducted during 2012–2015. A total of 922 patients with incident breast cancer (cases) and 913 hospital controls were recruited and interviewed using a standard questionnaire including information on eating behavior during both daytime and nighttime. We collected the timing, duration, types and frequencies of food intake of eating at nighttime. Odds ratios (ORs) for the risk of breast cancer in relation to nighttime eating-related variables were calculated by unconditional multivariable logistic regression.

**Results:**

Eating at night after 10 pm was significantly associated with breast cancer with an adjusted OR of 1.50 (95% confidence interval (CI) 1.06–2.12, *P* = 0.02), and the associations were stronger in women who had the longest duration of nighttime eating (≥20 years) (adjusted OR = 2.28 (95% CI 1.13–4.61, *P* = 0.02) and who ate late (midnight to 2 a.m.) (adjusted OR = 2.73, 95% CI 1.01–6.99, *P* = 0.04). Interestingly, nighttime eating was only associated with breast cancer among women who consumed staple foods (OR = 2.16, 95% CI 1.42–3.29, *P* < 0.001) but not those who ate vegetables or fruits as nighttime meals. The significant association between nighttime eating and breast cancer was observed among women with body mass index (BMI) <25 (OR = 2.29, 95% CI 1.48–3.52, *P* < 0.001) but not among women with BMI ≥25.

**Conclusions:**

Results from this study suggest a possible association between nighttime eating behavior and breast cancer. These findings need to be confirmed by independent large studies.

**Electronic supplementary material:**

The online version of this article (doi:10.1186/s13058-017-0821-x) contains supplementary material, which is available to authorized users.

## Background

Breast cancer is the most common cancer among women worldwide including women in Hong Kong [1, 2]. Although the age-standardized incidence of female breast cancer in Hong Kong is still lower compared to Northern America and Europe, it has been increasing sharply in recent decades [1]. Such an accelerating rate among Hong Kong women has been mostly attributed to changes in environmental exposure, particularly the adoption of a westernized lifestyle.

Night-eating behavior has become more common in recent years [3]. Evidence from experimental studies showed that rats fed in conflict with their natural nocturnal schedule gained weight despite no change in their diet, whereas there was no weight gain observed in the control group in which rats were fed at the normal time [4]. Epidemiologic studies have provided supportive evidence that food intake in the evening, especially closer to sleep, is more likely to lead to obesity than food consumption at other times of a day [5, 6].

Nighttime eating characterized by a circadian delay in daily food intake has been associated with alterations in neuroendocrine functions, including food regulatory proteins such as leptin, ghrelin and insulin, and circadian melatonin and cortisol hormones [7–9]. Food intake also functions as an important Zeitgeber (external cue that function to entrain biological rhythms) for peripheral clocks that orchestrate synchrony with brain clocks following the normal day-night cycle [10, 11]. Eating outside of the orchestrated synchronization has been shown to result in a phase shift and misalignment of normal daily circadian oscillations in rodents, showing a subsequent alteration of metabolic hormone concentrations, which may lead to obesity-related diseases such as cancer [12, 13].

Data on the link between night-eating behavior and breast cancer risk are sparse, other than a few experimental studies. Results from the USA study, the National Health and Nutrition Examination Survey (NHANES) showed that an increase in nighttime fasting duration was associated with improved glycemic regulation, which may relate to a reduced risk of breast cancer [14]. The goal of this study was to evaluate the association between night-eating behavior and female breast cancer risk in a breast cancer case-control study that included a comprehensive collection of risk factor data.

## Methods

Details of the study population have been previously described [15, 16]. Briefly, all participants were recruited from three local hospitals in Hong Kong between August 2012 and March 2015. The inclusion criteria for case participants were: (1) female, aged 20–84 years; (2) incident, histologically confirmed primary breast cancer (International Classification of Disease, Tenth Revision, code 50) diagnosed no more than 3 months prior to the recruitment interview; and (3) of Chinese ethnicity and residing in Hong Kong for at least 5 years. Patients were excluded if they were: (1) too young (<20 years old) or too old (≥85 years old); (2) patients who had been diagnosed more than 3 months prior to the date of interview or patients with recurrent breast cancer after initial treatment; and (3) had a history of cancer at any site before breast cancer diagnosis.

The inclusion criteria for control participants were: (1) Chinese women aged 20–84 years old; (2) no history of cancer; (3) admission to the same hospital during the same time period as the case participants; (4) frequency-matched by age (5-year age interval) to case participants; and (5) residents of Hong Kong for at least 5 years. Controls were excluded if they had physician-diagnosed cancer at any site. For our controls we selected patients who had a broad spectrum of diagnoses (such as diseases of the circulatory, genitourinary or nervous system) that were unrelated to breast cancer, to reduce or cap any possible bias [17, 18]. The current report consisted of 922 case participants and 913 controls. The study protocol was approved by both the Joint Chinese University of Hong Kong-New Territories East Cluster Clinical Research Ethics Committees and the Kowloon West Cluster. Written informed consent was obtained from both case participants and controls prior to the interview.

Face-to-face interviews were conducted with both case participants and controls, by trained interviewers using a standardized questionnaire. In addition to diet, other major variables included sociodemographic characteristics, smoking and alcohol drinking, reproductive factors, family cancer history, physical activity, sleeping habits and occupational history including shift work. Anthropometric risk factors were also recorded; height and weight were self-reported, whereas waist, hip circumference and subcutaneous fat thickness were measured by our interviewers using standard measurement tools. Details of recreational physical activities, including type (walking, hiking, running, swimming, ball games, *QiGong* or others), frequency and duration of each physical activity were recorded. In addition, information on commute including transportation methods and total time spent were also recorded in our questionnaire. Shift work was recorded and defined as “ever worked rotating shifts or night shift (from midnight to 5 a.m.) more than once a month for more than 1 year”. We also collected detailed data on nightshift work including type of shiftwork, frequency, duration, cumulative night shifts, and rotating schedules. Sleeping habits, including sleeping duration and quality in the last 5 years, were also collected in the questionnaire.

We assessed each participant’s overall dietary habits and supplement intakes within 5 years prior to the interview, using a reduced version of the Block food frequency questionnaire (FFQ) of National Cancer Institute (NCI) with a slight modification [19]. If the dietary habits had changed substantially during the past 5 years, the habits preceding the changes were used for analysis. Participants were asked about their dietary habits related to the frequency of consuming cereal (wheat, rice), coarse grain (e.g. maize, sorghum, millet), fresh vegetables including both green vegetables (e.g. cabbage, spinach, broccoli) and orange vegetables (e.g. carrot, tomato), fresh meat, fresh fruits, preserved vegetables, preserved meat, deep-fried foods, dairy products, soy products, tea and coffee. Daily dietary supplement intakes (e.g. vitamins, beta-carotene, calcium, etc.) were also recorded.

As nighttime food intake was not included in the NCI questionnaire, we developed specific questions to collect data on nighttime eating behavior. In order to avoid “double count”, we collected details of the type of food, timing of eating and duration of nighttime eating, instead of food frequency, which was already collected in the FFQ. Information was collected on nighttime eating and working history. For those who reported to have worked in one or more full-time jobs, a complete work history was recorded including night work/shift work status and nighttime eating behavior. Nighttime eating was defined as habitual food intake after 10 p.m. at least one time per week for more than one year. Among women who reported a history of nighttime eating, only 15% ate at nighttime less than once a week. Similar results were observed in the sensitivity analysis removing these women and therefore we used nighttime eating once or more per week as the cutoff point. Type of food, timing of eating (10 p.m.–12 a.m., 12 a.m.–2 a.m., 2 a.m.–4 a.m.) and duration (years) were recorded for those who reported having habitual nighttime eating. Meanwhile, for those who have never had a full-time job, nighttime eating habits within the most recent 5-year period and details of the frequency of nighttime eating and the food consumed were recorded. Detailed exposure assessment questions about nighttime eating can be found in Additional file 1.

Nighttime food intake was classified into two categories, staple foods and snacks, by meal size and type of food. Staple foods referred to a regular meal, which often contained one type of food that was rich in carbohydrate (e.g. wheat, rice), in addition to variable vegetable or meat content; snacks referred to a portion of food smaller than a regular meal, often containing one or more items such as nuts, dairy products, bakery food, desserts or chips. Those who reported eating multiple food items including both staple foods and snacks were categorized as staple-food eaters. Within each category, food items were further classified into several different categories: noodles (including wheat noodles, rice noodles, instant noodles and pasta), rice (steamed rice and congee), baked goods (breads, cakes, biscuits, etc.), vegetables (as the main ingredient), and meat (as a primary ingredient).

The independent *t* test and chi-square test were performed to test the differences between case participants and controls in sociodemographic factors for continuous and categorical data, respectively. Multivariable logistic regression was used to calculate the odds ratio (OR) and 95% confidence interval (95% CIs) for the association between variables related to nighttime eating and breast cancer risk, with adjustment for potential confounding factors. To select potential confounders to be included in the multivariable model, we first conducted univariate analysis with adjustment for age (age at diagnosis in case participants and age at interview in controls) and a single risk factor only.

All known or suspected breast cancer risk factors ascertained, including detailed shift work, were analyzed in the univariate analysis, and those with *P* values <0.05 were further included in the final multivariable logistic regression models. The factors assessed included sociodemographic characteristics, reproductive factors, family history of cancer, history of benign breast disease and other medical conditions, smoking and alcohol consumption, anthropometric factors, physical activity, lifetime work history and shift work status, sleep pattern and exposure to light at night.

Three multivariable regression models were presented separately, adjusted for: (1) age only; (2) age and all breast cancer risk factors with *P* < 0.05 in the univariate analysis; and (3) model (1) plus total dietary intake variables that were significantly associated with breast cancer. To evaluate the potential effect modification, we formally tested the interactions between nighttime eating and menopausal status, estrogen receptor (ER) status and body mass index (BMI) by including interaction terms in the regression models involving all subjects. We also conducted stratified analyses of these variables.

We performed a number of sensitivity analyses including analysis (1) excluding controls with digestive system disease (which may be associated with dietary intake or nighttime eating) to probe the potential bias caused by medical diseases in hospital-based controls; (2) analyzing a specific group of patients who underwent breast biopsy and were initially considered as patients with breast cancer but were eventually confirmed to be non-cancer case participants; and (3) removing women who reported particular frequencies of nighttime eating to find an appropriate cutoff point for nighttime eating behavior.

## Results

The basic characteristics of case participants with breast cancer and the distribution of selected breast cancer risk factors are presented in Table 1. The mean age at breast cancer diagnosis was 56.0 ± 11.8 years. Compared with controls, case participants were significantly younger at menarche and older at first birth, were more likely to have family history of cancer in first-degree relatives, were more likely to be obese (BMI >25) and were less likely to have ever been employed in shift work. Other variables including menopausal status, educational attainment, the use of oral contraceptives or hormone replacement therapy, parity, smoking and alcohol drinking and sleeping pattern did not vary significantly by case-control status.

**Table 1:** Distribution of basic characteristics and selected breast cancer risk factors among Hong Kong Chinese women, 2012–15

Variables	Case participants (*n* = 922)	Controls (*n* = 913)
Age at interview (years)	56.0 ± 11.8	53.7 ± 11.7
Age at menarche (years)	13.6 ± 2.0	13.8 ± 2.3
Age at first birth (years)	26.4 ± 4.9	25.6 ± 4.7
BMI (kg/m^2^)	23.5 ± 3.7	23.0 ± 3.9
BMI^a^		
Underweight (<18.5)	44 (4.8)	74 (8.1)
Normal (18.5–22.9)	329 (35.7)	350 (38.3)
Overweight (23.0–24.9)	160 (17.4)	157 (17.2)
Obesity I (25–29.9)	205 (22.2)	162 (17.7)
Obesity II (≥30)	45 (4.9)	40 (4.4)
Unknown	139 (15.1)	130 (14.2)
First-degree family cancer history		
Yes	323 (35.0)	246 (26.9)
No	572 (62.0)	652 (71.4)
Unknown	27 (2.9)	15 (1.6)
Family history of breast cancer		
Yes	70 (7.6)	28 (3.1)
No	825 (89.5)	870 (95.3)
Unknown	27 (2.9)	15 (1.6)
Parity, number of births		
0	164 (17.8)	154 (16.8)
1–2	484 (52.5)	473 (51.8)
≥3	228 (24.7)	269 (29.5)
Unknown	46 (5.0)	17 (1.9)
Smoking		
Ever	63 (6.8)	69 (7.6)
Never	859 (93.2)	844 (92.4)
Alcohol drinking		
Yes	47 (5.1)	41 (4.5)
No	875 (94.9)	872 (95.5)
Shift work		
Yes	111 (12.0)	150 (16.4)
No	771 (83.6)	737 (80.7)
Unknown	40 (4.3)	26 (2.8)
Histological type		
Ductal	809 (87.7)	
Lobular	30 (3.3)	
Mucinous	36 (3.9)	
Medullary	3 (0.3)	
Others	44 (4.8)	
Tumor stage		
Stage I	240 (31.8)	
Stage II	298 (39.4)	
Stage III	96 (12.7)	
Stage IV	42 (5.6)	
Un-staged	79 (10.5)	
Estrogen receptor status		
Positive	601 (76.7)	
Negative	183 (23.3)	
Progesterone receptor status		
Positive	450 (59.5)	
Negative	306 (40.5)	
C-erbB2/HER2		
Negative(IHC score 0/1)	351 (45.8)	
Weakly positive (IHC score 2)	268 (35.0)	
Positive (IHC score 3)	147 (19.2)	

Values expressed as mean ± SD or number (percent). Subjects with missing values were excluded from the analyses. ^a^Cutoff points for body mass index (BMI) recommended by the World Health Organization to classify overweight and obese individuals in Asian populations. *HER2* human epidermal growth factor receptor 2, *IHC* immunohistochemical analysis

The frequency of total food consumption among case participants and controls in the 5 years prior to the study is shown in Additional file 2: Table S1. After adjusting for known breast cancer risk factors including age at interview, age at menarche, age at first birth, BMI, family history of any cancer among first-degree relatives, shift work and all major food categories, case participants with breast cancer tended to have higher consumption of deep-fried food (OR = 1.68, 95% CI 1.08–2.59, *P* = 0.02) and dairy products (OR = 1.37, 95% CI 1.00–1.87, *P* = 0.05). The intake of other food items did not differ significantly in case participants and controls (Additional file 2: Table S1).

Associations between nighttime eating habits and breast cancer are summarized in Table 2. Nighttime eating was more common among case participants with breast cancer (17.4%) than among controls (15.2%). After adjusting for selected confounding factors (including dietary factors), we observed a significant association between risk of breast cancer among case participants who had a habit of nighttime eating compared with those who never ate at night (OR = 1.50, 95% CI 1.06–2.12, *P* = 0.02). The association was stronger among women who consumed staple foods (adjusted OR = 2.16, 95% CI 1.42–3.29, *P* < 0.001), particularly noodles (OR = 2.79, 95% CI 1.58–4.94, *P* < 0.001) or rice (OR = 2.58, 95% CI 1.42–4.69, *P* = 0.002). Breast cancer in case participants may be associated with consumption of meat at night time (OR = 1.73, 95% CI 0.84–3.59, *P* = 0.14), but this was not statistically significant. There was no evidence of a relationship between breast cancer and the consumption of vegetables (OR = 1.14, 95% CI 0.63–2.55, *P* = 0.61) or fruits (OR = 1.20, 95% CI 0.51–2.83, *P* = 0.68) at nighttime.

**Table 2:** Associations between nighttime eating behavior and breast cancer in Hong Kong Chinese women

Variables	Case participants (*n* = 922)	Controls (*n* = 913)	OR^a^ (95% CI)	OR^b^ (95% CI)	OR^c^ (95% CI)	*P* value^d^
Nighttime eating after 10 p.m.						
Never^e^	762 (82.6)	774 (84.8)	1.00 (ref)	1.00 (ref)	1.00 (ref)	
Ever	160 (17.4)	139 (15.2)	1.31 (1.02–1.68)	1.51 (1.08–2.12)	1.50 (1.06–2.12)	*0.02*
Meal type						
Never^e^	762 (82.6)	774 (84.8)	1.00 (ref)	1.00 (ref)	1.00 (ref)	
Staple food	123 (13.3)	86 (9.4)	1.65 (1.22–2.22)	2.16 (1.43–3.28)	2.16 (1.42–3.29)	<*0.001*
Snacks	37 (4.0)	53 (5.8)	0.75 (0.48–1.15)	0.75 (0.44–1.29)	0.74 (0.43–1.27)	0.27
Food type						
Never^e^	762 (82.6)	774 (84.8)	1.00 (ref)	1.00 (ref)	1.00 (ref)	
Noodles	70 (7.6)	40 (4.4)	1.93 (1.29–2.90)	2.78 (1.58–4.87)	2.79 (1.58–4.94)	<*0.001*
Rice	62 (6.7)	37 (4.1)	1.90 (1.24–2.91)	2.53 (1.40–4.47)	2.58 (1.42–4.69)	0.*002*
Baked goods	30 (3.3)	37 (4.1)	0.88 (0.54–1.44)	1.27 (0.71–2.28)	1.19 (0.66–2.16)	0.56
Meat	31 (3.4)	23 (2.9)	1.48 (0.85–2.57)	1.89 (0.92–3.88)	1.73 (0.84–3.59)	0.14
Vegetable	24 (2.6)	22 (2.4)	1.09 (0.76–2.11)	1.15 (0.67–2.67)	1.14 (0.63–2.55)	0.61
Fruit	15 (1.6)	13 (1.4)	1.22 (0.58–2.59)	1.18 (0.51–2.74)	1.20 (0.51–2.83)	0.68

^a^Adjusted for age at interview. ^b^Adjusted for age at interview, age at menarche, age at first birth, body mass index (BMI), history of cancer in a first-degree family member and shift work. ^c^Adjusted for age at interview, age at menarche, age at first birth, BMI, history of cancer in a first-degree family member, shift work and other dietary factors (consumption of cereals, deep-fried foods, preserved meats and dairy products). ^d^ *P* values from model 3. ^e^Those who did not have habitual nighttime eating behavior. *P* values in italics are statistically significant. *ref* reference

We further conducted a more detailed analysis of the 894 case participants and 882 control participants who had completed records on duration and timing of nighttime eating habits (Table 3). Long duration of nighttime eating (>20 years) appeared to have the strongest association with breast cancer status (OR = 2.28, 95% CI 1.13–4.61, *P* = 0.02). The association with nighttime eating did not seem to vary significantly by the timing of food consumption, although eating between midnight and 2 a.m. had the strongest effect in the multivariable model (OR = 2.73, 95% CI 1.01–6.99, *P* = 0.04).

**Table 3:** Associations between breast cancer and the timing of eating and duration of nighttime eating behavior in Hong Kong Chinese women

Variables	Case participants (*n* = 894)	Controls (*n* = 882)	OR^a^ (95% CI)	OR^b^ (95% CI)	OR^c^ (95% CI)	*P* value^d^
Duration						
None	762 (85.2)	774 (87.8)	1.00 (ref)	1.00 (ref)	1.00 (ref)	
<10	48 (5.4)	45 (5.1)	1.34 (0.87–2.06)	1.76 (0.99–3.13)	1.75 (0.97–3.14)	0.06
10–19	40 (4.5)	42 (4.8)	1.05 (0.67–1.64)	1.46 (0.81–2.63)	1.46 (0.80–2.65)	0.22
≥20	44 (4.9)	21 (2.4)	2.23 (1.30–3.82)	2.08 (1.05–4.15)	2.28 (1.13–4.61)	*0.02*
Timing						
None	762 (85.2)	774 (87.8)	1.00 (ref)	1.00 (ref)	1.00 (ref)	
10 p.m.–12 a.m.	78 (8.7)	58 (6.6)	1.54 (1.08–2.21)	1.80 (1.11–2.90)	1.84 (1.14–2.98)	*0.01*
12 a.m.–2 a.m.	20 (2.2)	15 (1.7)	1.43 (0.72–2.82)	2.50 (1.00–6.26)	2.73 (1.01–6.99)	*0.04*
2 a.m.–4 a.m.	12 (1.3)	12 (1.4)	1.08 (0.48–2.43)	1.98 (0.79–4.97)	1.93 (0.76–4.94)	0.17
Irregular^e^	22 (2.5)	23 (2.6)	1.14 (0.62–2.08)	0.99 (0.42–2.37)	0.99 (0.41–2.40)	0.98

^a^Adjusted for age at interview. ^b^Adjusted for age at interview, age at menarche, age at first birth, body mass index (BMI), history of cancer in a first-degree family member and shift work. ^c^Adjusted for age at interview, age at menarche, age at first birth, BMI, history of cancer in a first-degree family member, shift work and other dietary factors (consumption of cereals, deep-fried foods, preserved meats and dairy products). ^d^ *P* value for model 3. ^e^No fixed time for nighttime eating. *P* values in italics are statistically significant. *ref* reference

Results of analyses stratified by menopausal status and ER status are presented in Additional files 3 and 4. Nighttime associations were similar in premenopausal and postmenopausal women overall and in subgroup analyses by meal type (Additional file 3: Table S2). Similar results were also observed among women with ER-positive and ER-negative cancer, with stronger associations seen among women with ER-positive compared with ER-negative cancer (Additional file 4: Table S3), possibly due to the small number of ER-negative case participants.

Table 4 shows the analyses stratified by BMI (BMI <25 and BMI ≥25). The association between nighttime eating and breast cancer was only observed among women with BMI <25 (OR = 2.29, 95% CI 1.48–3.52, *P* < 0.001) but not among women with BMI ≥25 (OR = 0.65, 95% CI 0.36–1.18, *P* = 0.16). A similar pattern was observed in subgroup analyses by meal and food type (Table 4). The interaction between nighttime eating and BMI was statistically significant (*P* for interaction = 0.01 particularly for consumption of staple food (*P* for interaction = 0.03). Similar results were obtained when a different cutoff (BMI = 23) was used (data not shown).

**Table 4:** Associations between nighttime eating and breast cancer stratified by BMI

Variables	BMI <25			BMI ≥25			Interaction by BMI status
	Case participants (*n* = 533)	Controls (*n* = 581)	OR (95% CI)^a^	Case participants (*n* = 250)	Controls (*n* = 202)	OR (95% CI)^a^	
Nighttime eating after 10 p.m.							
Never	434	502	1.00 (ref)	205	158	1.00 (ref)	
Ever	99	79	2.29 (1.48–3.52)*	45	44	0.65 (0.36–1.18)	*0.01*
Meal type							
Never	434	502	1.00 (ref)	205	158	1.00 (ref)	
Staple food	81	51	3.30 (1.95–5.59)*	34	25	0.85 (0.40–1.79)	*0.03*
Snacks	23	30	1.26 (0.62–2.57)	12	20	0.38 (0.16–0.93)*	0.20
Food type							
Never	434	502	1.00 (ref)	205	158	1.00 (ref)	
Noodles	44	26	4.07 (2.02–8.20)*	20	10	1.01 (0.35–2.95)	0.27
Rice	39	20	4.41 (2.05–9.49)*	15	13	0.85 (0.30–2.36)	0.09
Baked goods	21	23	1.81 (0.88–3.73)	8	9	0.46 (0.15–1.38)	0.21
Meat	20	12	3.14 (1.25–7.88)*	9	9	0.51 (0.14–1.86)	0.12
Vegetable	14	12	1.68 (0.64–4.38)	10	7	0.88 (0.22–3.57)	0.81
Fruit	10	7	2.98 (1.02–8.72)*	1	6	0.13 (0.01–1.20)	0.08

^a^Adjusted for age at interview, age at menarche, age at first birth, first-degree family cancer history, shift work and other dietary factors (consumption of cereals, deep-fried foods, preserved meats and dairy products)

*Statistically significant (*P* value <0.05). For “Interaction by BMI status”, *P* values in italics are statistically significant. *BMI* body mass index, *ref* reference

Similar results were obtained from the sensitivity analysis excluding controls with digestive disease (data not shown). Furthermore, to evaluate the influence of recall or interview bias, we compared the proportion of patients who reported nighttime eating among a group of 131 patients, who were initially suspected of having breast cancer but eventually were confirmed to have benign breast disease, to all controls and observed similar frequencies of nighttime eating (16.0% vs. 15.2%, respectively).

## Discussion

In this breast cancer case-control study among Chinese women in Hong Kong, we showed that nighttime eating might be significantly associated with risk of breast cancer, in particular among women with BMI <25, who have had nighttime eating habits for more than 20 years and who consume energy-dense foods rich in carbohydrate after 10 p.m. (such as rice or noodles), implicating nighttime eating as a potential new risk factor for breast cancer.

Nighttime eating has long been reported to have a negative impact on health and body composition, with consequences that include weight gain and obesity, which are well-known risk factors for postmenopausal breast cancer [6]. Animal studies and epidemiologic data have provided convincing evidence that consuming a larger proportion of calories later in the day as opposed to earlier in the day is associated weight gain and obesity [5, 20–24]. In particular, obesity is more prevalent among people with night eating syndrome (NES), which is an eating disorder characterized by a delayed circadian pattern of food intake [25]. However, studies on how nighttime eating behavior influences the risk of breast cancer are very limited. Our results are consistent with previous findings based on data from the NHANES and the Women’s Healthy Eating and Living study, in which longer nighttime fasting was associated with reduced risk of breast cancer and recurrence of breast cancer [14, 26]. In addition, our findings related to food choice are also in line with previous literature reporting that only the intake of large quantities of high-energy food, and not small low-energy snacks, at night had a negative health impact [27–32].

The mechanisms underlying the association between nighttime eating and risk of breast cancer remains unknown and may be mediated through weight gain and obesity [5, 21–24], glucose tolerance and insulin resistance [30, 33, 34], increased systemic inflammation [35], and circadian rhythm disruption caused by food intake [36, 37]. Interestingly, we only observed the association between nighttime eating and breast cancer among slim women, suggesting that the association might be mediated through an obesity-independent mechanism. The strong association between obesity and breast cancer may have masked the association between nighttime eating and breast cancer among obese women. Alternatively, it is also possible that the lack of association among obese women is driven by the underreporting of nighttime food consumption in obese case participants as this has been shown to be a common issue [38, 39]. In fact, case participants with breast cancer were indeed more likely to be obese (27.1% for case participants vs. 22.1% for controls) in our study and the potential underreporting would have biased the results toward the null.

Consistent with a recent large meta-analysis [38] and two Chinese studies with a prospective cohort study design [39, 40], night shift work was not significantly associated with increased risk of breast cancer in our study, suggesting that the association between nighttime eating and breast cancer was not driven by the night shift work. Circadian disruption caused by nighttime eating may also lead to disturbances in the quantity and quality of sleep [41], which may adversely impact health, including risk of breast cancer [41, 42]. However, studies on associations between sleeping duration, quality or disturbance and breast cancer risk have yielded inconsistent or even conflicting findings [43–49]. In our study, sleeping habits (such as duration and quality) did not differ between case participants and controls. Further, the association between nighttime eating and breast cancer seem to be restricted to specific food types and to lean women, suggesting that the association was not driven by sleep disturbance.

This study provides new insights into nighttime eating habits as potential risk factors for breast cancer. However, we are aware that our study is limited by the hospital case-control design and the small number of subjects, and therefore our findings need to be interpreted with caution. Large prospective studies are needed to confirm these results. Nevertheless, we conducted detailed analyses of nighttime eating variables such as timing, duration, frequency, and type of food consumption, with careful adjustment for known breast cancer risk factors, total dietary habits and night shift work.

We also conducted several sensitivity analyses to assess whether the associations were influenced by recall bias or the potential issue related to self-reported dietary habits among hospital controls. In order to minimize recall bias, we recruited only case participants with incident cancer and tried to introduce this study to participants as a general “women’s health” study rather than a cancer study. Analysis of test-re-test reliability was also conducted to check the reproducibility of interview results. Telephone interviews were conducted by the same interviewer at least one month after the first interview with 158 case participants (17.1%) and 153 controls (16.8%). There was good agreement for identification of nighttime eating exposure (consistency rate = 83%; kappa = 0.59, 95% CI 0.35–0.83) between the first and second interviews. In addition, in sensitivity analysis comparing 131 patients, who were initially suspected of having breast cancer but eventually were confirmed to have benign breast disease, to all controls also suggested that recall or interviewer bias may not have seriously influenced our results. Further, dietary habits in hospital controls may differ from those of the general population due to health conditions, consciousness or diseases, particularly among those with diseases of the digestive system. However, previous methodology studies showed that even if a particular segment of the controls has altered diet practices, bias is constrained [18]. In support of this, we recruited hospital controls with a broad range of diagnoses, and the sensitivity analyses indicated that the association between nighttime eating and breast cancer remained significant after excluding control subjects with digestive disease.

Due to the retrospective study design, data on quantities of food intake or total calorie intake were not collected due to difficulty in accurately recalling the amounts of food consumed a long time ago. To address this limitation, we accounted for the overall consumption of different types of food items and BMI in our analyses. Further, we found that the association between nighttime eating and breast cancer was more significant among slim women, suggesting that the association was not driven by excessive calories.

The FFQ is a tool to estimate food and nutrient consumption and has been widely used in investigating the associations between diet and chronic disease [50]. However, the FFQ may be sensitive to the diverse lifestyle, eating habits and dietary preferences in the population concerned [51]. In this study, we used NCI questionnaire to access the dietary intake and a few modifications had been made in relation to dietary habits in the Hong Kong Chinese population. Although this questionnaire have been reported to perform well in many populations [52], the reproducibility and validity of this questionnaire should be conducted among the Hong Kong population in future studies.

## Conclusion

In conclusion, our findings suggest that nighttime eating was more common among breast cancer case participants compared with control participants. More epidemiological studies, especially with a prospective design and a large number of subjects, are needed to confirm this association and to explore the underlying mechanisms.

## Additional files

Additional file 1: Nighttime eating exposure assessment. (DOCX 13 kb)
Additional file 2: Table S1. Distribution of dietary factors among breast cancer cases and controls in Hong Kong Chinese women, 2012–15. (DOCX 32 kb)
Additional file 3: Table S2. Associations between nighttime eating and breast cancer stratified by menopausal status. (DOCX 14 kb)
Additional file 4: Table S3. Associations between nighttime eating and breast cancer stratified by ER status. (DOCX 14 kb)

## Abbreviations

BMI: Body mass index
CI: Confidence interval
ER: Estrogen receptor status
FFQ: Food frequency questionnaire
HER2: Human epidermal growth factor receptor 2
IHC: Immunohistochemical analysis
NCI: National Cancer Institute
NES: Night eating syndrome
NHNES: National Health and Nutrition Examination Survey
OR: Odds ratio
