# Supplementary material for: Nighttime eating and breast cancer among Chinese women in Hong Kong
Source: Breast Cancer Res. 2017 Mar 17;19:31. doi: 10.1186/s13058-017-0821-x (PMC5356318; doi:10.1186/s13058-017-0821-x)
Supplement: Additional file 1: — Nighttime eating exposure assessment. (DOCX 13 kb) [file 13058_2017_821_MOESM1_ESM.docx]

Additional File 1

Nighttime eating exposure assessment

(A) For those who reported working full-time (≥30 hours per week for at least 1 year):

1. For each full-time job you have done, did you have a habit of eating any food after 10pm at least one time per week?
2. If yes, when did you usually eat the food? Please specify the timing.
3. What’s the most common food you chose to eat? Please specify food names in each category.

(1) Staple food (such as rice, noodle, meat);

(2) Snacks (such as nuts, chips, etc.).

1. How long do you do this job and have the habit of nighttime eating?

(B) For those who have never done a full-time job:

1. In the most recent five years, did you have a habit of eating any food after 10pm?
2. How often did you eat at night?

(1) Never; (2) Less than once per year; (3) Less than once per month; (4) 1-3 times per month; (5) 1-3 times per week; (6) 4-6 times per week; (7) Every day.

1. What’s the most common food that you chose to eat? Please specify food names in each category.

(1) Staple food (such as rice, noodle, meat);

(2) Snacks (such as nuts, chips, etc.).
