# Supplementary material for: Nighttime eating and breast cancer among Chinese women in Hong Kong
Source: Breast Cancer Res. 2017 Mar 17;19:31. doi: 10.1186/s13058-017-0821-x (PMC5356318; doi:10.1186/s13058-017-0821-x)
Supplement: Additional file 2: Table S1. — Distribution of dietary factors among breast cancer cases and controls in Hong Kong Chinese women, 2012–15. (DOCX 32 kb) [file 13058_2017_821_MOESM2_ESM.docx]

Additional File 2

Table S1 Distribution of dietary factors among breast cancer cases and controls in Hong Kong Chinese women, 2012-15

| Food intake frequency | Cases  (n=922) |  |  | Controls  (n=913) |  | *P value* ^a^ | OR^b^  (95%CI) | OR^c^  (95%CI) | OR^d^  (95%CI) | *P* value ^e^ |
| --- | --- | --- | --- | --- | --- | --- | --- | --- | --- | --- |
| Cereals |  |  |  |  |  | **0.006** |  |  |  |  |
| ≤1 per day | 85 (9.2) |  |  | 103 (11.3) |  |  | 1.00 (ref) | 1.00 (ref) | 1.00 (ref) |  |
| 2 per day | 579 (62.8) |  |  | 506 (55.4) |  |  | 1.38 (1.01-1.89)* | 1.51 (1.00-2.30)* | 1.45 (0.94-2.24) | 0.10 |
| ≥3 per day | 258 (28.0) |  |  | 304 (33.3) |  |  | 1.02 (0.73-1.43) | 1.17 (0.75-1.82) | 1.15 (0.72-1.82) | 0.57 |
| Coarse grains |  |  |  |  |  | 0.83 |  |  |  |  |
| <1 per month | 117 (12.7) |  |  | 120 (13.1) |  |  | 1.00 (ref) | 1.00 (ref) | 1.00 (ref) |  |
| <1 per week | 263 (28.5) |  |  | 253 (27.7) |  |  | 1.09 (0.80-1.49) | 1.25 (0.83-1.87) | 1.28 (0.83-1.95) | 0.26 |
| 1-3 per week | 379 (41.1) |  |  | 365 (40.0) |  |  | 1.10 (0.82-1.48) | 1.10 (0.74-1.62) | 1.19 (0.78-1.81) | 0.42 |
| ≥3 per week | 163 (17.7) |  |  | 175 (19.2) |  |  | 0.93 (0.66-1.30) | 0.94 (0.61-1.45) | 1.09 (0.68-1.74) | 0.73 |
| Fresh green vegetables |  |  |  |  |  | 0.18 |  |  |  |  |
| <1 per day | 200 (21.7) |  |  | 175 (19.2) |  |  | 1.00 (ref) | 1.00 (ref) | 1.00 (ref) |  |
| ≥1 per day | 722 (78.3) |  |  | 738 (80.8) |  |  | 0.86 (0.68-1.08) | 0.82 (0.61-1.10) | 0.88 (0.65-1.21) | 0.44 |
| Fresh orange vegetables |  |  |  |  |  | 0.72 |  |  |  |  |
| <1 per week | 326 (35.4) |  |  | 324 (35.5) |  |  | 1.00 (ref) | 1.00 (ref) | 1.00 (ref) |  |
| 1-3 per week | 553 (60.0) |  |  | 539 (59.0) |  |  | 1.04 (0.85-1.26) | 0.91 (0.71-1.17) | 0.97 (0.74-1.28) | 0.83 |
| >3 per week | 43 (4.7) |  |  | 50 (5.5) |  |  | 0.90 (0.58-1.40) | 0.56 (0.31-1.00) | 0.68 (0.37-1.26) | 0.22 |
| Fresh meat |  |  |  |  |  | 0.10 |  |  |  |  |
| <1 per day | 81 (8.8) |  |  | 101 (11.1) |  |  | 1.00 (ref) | 1.00 (ref) | 1.00 (ref) |  |
| ≥1 per day | 841 (91.2) |  |  | 812 (88.9) |  |  | 1.40 (1.03-1.92)* | 1.36 (0.91-2.04) | 1.25 (0.81-1.91) | 0.32 |
| Fresh fruit |  |  |  |  |  | 0.38 |  |  |  |  |
| <1 per week | 175 (19.0) |  |  | 187 (20.5) |  |  | 1.00 (ref) | 1.00 (ref) | 1.00 (ref) |  |
| <1 per day | 476 (51.6) |  |  | 442 (48.4) |  |  | 1.19 (0.93-1.53) | 1.09 (0.80-1.50) | 0.97 (0.70-1.36) | 0.87 |
| ≥1 per day | 271 (29.4) |  |  | 284 (31.1) |  |  | 1.02 (0.78-1.33) | 0.92 (0.65-1.29) | 0.91 (0.63-1.32) | 0.63 |
| Deep-fried foods |  |  |  |  |  | **0.04** |  |  |  |  |
| <1 per year | 122 (13.2) |  |  | 152 (16.6) |  |  | 1.00 (ref) | 1.00 (ref) | 1.00 (ref) |  |
| <1 per week | 533 (57.8) |  |  | 533 (58.4) |  |  | 1.41 (1.07-1.85)* | 1.53 (1.08-2.19)* | 1.32 (0.90-1.95) | 0.16 |
| ≥1 per week | 267 (29.0) |  |  | 228 (25.0) |  |  | 1.83 (1.34-2.50)* | 1.75 (1.17-2.61)* | 1.68 (1.08-2.59)* | **0.02** |
| Preserved meats |  |  |  |  |  | **0.004** |  |  |  |  |
| <1 per year | 201 (21.8) |  |  | 260 (28.5) |  |  | 1.00 (ref) | 1.00 (ref) | 1.00 (ref) |  |
| <1 per month | 595 (64.5) |  |  | 533 (58.4) |  |  | 1.52 (1.22-1.90)* | 1.35 (1.02-1.79)* | 1.07 (0.77-1.48) | 0.68 |
| ≥1 per month | 126 (13.7) |  |  | 120 (13.1) |  |  | 1.47 (1.07-2.01)* | 1.34 (0.91-1.97) | 1.11 (0.72-1.70) | 0.63 |
| Preserved vegetables |  |  |  |  |  | 0.23 |  |  |  |  |
| <1 per year | 139 (15.1) |  |  | 159 (17.4) |  |  | 1.00 (ref) | 1.00 (ref) | 1.00 (ref) |  |
| <1 per month | 294 (31.9) |  |  | 254 (27.8) |  |  | 1.35 (1.01-1.80)* | 1.45 (1.00-2.10)* | 1.27 (0.83-1.95) | 0.26 |
| <1 per week | 328 (35.6) |  |  | 336 (36.8) |  |  | 1.15 (0.87-1.52) | 1.21 (0.85-1.73) | 1.09 (0.73-1.64) | 0.66 |
| ≥1 per week | 161 (17.5) |  |  | 164 (18.0) |  |  | 1.17 (0.85-1.61) | 1.12 (0.75-1.67) | 1.02 (0.66-1.60) | 0.92 |
| Dairy products |  |  |  |  |  | **0.03** |  |  |  |  |
| <1 per year | 315 (34.2) |  |  | 338 (37.0) |  |  | 1.00 (ref) | 1.00 (ref) | 1.00 (ref) |  |
| <1 per week | 269 (29.2) |  |  | 212 (23.2) |  |  | 1.46 (1.15-1.86)* | 1.48 (1.10-2.00)* | 1.37 (1.00-1.87)* | **0.05** |
| <1 per day | 222 (24.1) |  |  | 246 (26.9) |  |  | 0.99 (0.78-1.26) | 1.12 (0.83-1.50) | 1.08 (0.79-1.48) | 0.61 |
| ≥1 per day | 116 (12.6) |  |  | 117 (12.8) |  |  | 1.01 (0.74-1.36) | 1.11 (0.76-1.63) | 1.22 (0.81-1.83) | 0.34 |
| Soy products |  |  |  |  |  | 0.19 |  |  |  |  |
| <1 per year | 73 (7.9) |  |  | 91 (10.0) |  |  | 1.00 (ref) | 1.00 (ref) | 1.00 (ref) |  |
| <1 per week | 423 (45.9) |  |  | 390 (42.7) |  |  | 1.46 (1.04-2.06)* | 1.55 (0.98-2.47) | 1.40 (0.85-2.28) | 0.18 |
| ≥1 per week | 426 (46.2) |  |  | 432 (47.3) |  |  | 1.38 (0.98-1.94) | 1.40 (0.88-2.23) | 1.35 (0.83-2.21) | 0.23 |
| Tea |  |  |  |  |  | 0.68 |  |  |  |  |
| <1 per week | 381 (41.3) |  |  | 396 (43.4) |  |  | 1.00 (ref) | 1.00 (ref) | 1.00 (ref) |  |
| <1 per day | 166 (18.0) |  |  | 168 (18.4) |  |  | 1.04 (0.80-1.34) | 0.93 (0.67-1.29) | 0.87 (0.62-1.22) | 0.43 |
| ≥1 per day | 350 (38.0) |  |  | 332 (36.4) |  |  | 1.05 (0.86-1.30) | 0.98 (0.75-1.26) | 0.96 (0.73-1.25) | 0.75 |
| Unknown | 25 (2.7) |  |  | 17 (1.9) |  |  | − | − | − |  |
| Coffee |  |  |  |  |  | 0.63 |  |  |  |  |
| <1 per week | 658 (71.4) |  |  | 674 (73.8) |  |  | 1.00 (ref) | 1.00 (ref) | 1.00 (ref) |  |
| <1 per day | 77 (8.4) |  |  | 79 (8.7) |  |  | 1.02 (0.73-1.43) | 0.96 (0.63-1.45) | 0.88 (0.57-1.35) | 0.56 |
| ≥1 per day | 168 (18.2) |  |  | 153 (16.8) |  |  | 1.15 (0.90-1.47) | 0.99 (0.73-1.34) | 0.88 (0.64-1.21) | 0.42 |
| Unknown | 19 (2.1) |  |  | 7 (0.8) |  |  | − | − | − |  |
| Vitamins and supplements |  |  |  |  |  | 0.58 |  |  |  |  |
| No | 705 (76.5) |  |  | 696 (76.2) |  |  | 1.00 (ref) | 1.00 (ref) | 1.00 (ref) |  |
| Yes | 191 (20.7) |  |  | 201 (22.0) |  |  | 0.91 (0.72-1.14) | 0.83 (0.63-1.09) | 0.84 (0.63-1.12) | 0.23 |
| Unknown | 26 (2.8) |  |  | 16 (1.8) |  |  | − | − | − |  |

^a^ P values were obtained using the Chi-square test comparing breast cancer cases to controls.

^b^ Adjusted for age at interview.

^c^ Adjusted for age at interview, age at menarche, age at first birth, body mass index and first-degree family cancer history (yes, no), shift work (yes, no).

^d^ Adjusted for age at interview, age at menarche, age at first birth, body mass index and first-degree family cancer history (yes, no), shift work (yes, no), and other dietary factors in this table.

^e^ *P* value from model d.

* Significant level *P* value <0.05.
