# Supplementary material for: Nighttime eating and breast cancer among Chinese women in Hong Kong
Source: Breast Cancer Res. 2017 Mar 17;19:31. doi: 10.1186/s13058-017-0821-x (PMC5356318; doi:10.1186/s13058-017-0821-x)
Supplement: Additional file 3: Table S2. — Associations between nighttime eating and breast cancer stratified by menopausal status. (DOCX 14 kb) [file 13058_2017_821_MOESM3_ESM.docx]

Additional File 3

Table S2 Associations between nighttime eating and breast cancer risk stratified by menopausal status

| Variables | Pre-menopausal | | |  | Post-menopausal | | |
| --- | --- | --- | --- | --- | --- | --- | --- |
|  | Cases  (n=336) | Controls  (n=354) | OR(95% CI)^a^ |  | Cases  (n=583) | Controls  (n=559) | OR(95% CI)^a^ |
| Nighttime eating after 10pm |  |  |  |  |  |  |  |
| Never | 261 | 276 | 1.00(Ref) |  | 498 | 498 | 1.00(Ref) |
| Ever | 75 | 78 | 1.48 (0.86-2.53) |  | 85 | 61 | 1.58 (0.99-2.52) |
| Meal type |  |  |  |  |  |  |  |
| Never | 261 | 276 | 1.00(Ref) |  | 498 | 498 | 1.00(Ref) |
| Staple food | 60 | 50 | 1.97 (1.03-3.74)* |  | 63 | 36 | 2.37 (1.33-4.24)* |
| Snacks | 18 | 32 | 0.87 (0.38-2.03) |  | 24 | 25 | 0.79 (0.38-1.64) |
| Food type |  |  |  |  |  |  |  |
| Never | 261 | 276 | 1.00(ref) |  | 498 | 498 | 1.00(ref) |
| Noodles | 32 | 27 | 1.65 (0.75-3.63) |  | 38 | 13 | 4.88 (2.00-11.9)* |
| Rice | 32 | 18 | 3.17 (1.23-8.20)* |  | 30 | 19 | 2.16 (0.97-4.82) |
| Baked goods | 13 | 19 | 1.29 (0.49-3.42) |  | 17 | 18 | 1.25 (0.58-2.69) |
| Meat | 18 | 8 | 6.27 (1.55-25.47)* |  | 13 | 15 | 0.71 (0.26-1.97) |
| Vegetable | 13 | 3 | NA |  | 11 | 14 | 0.73 (0.24-2.23) |
| Fruit | 4 | 7 | 0.57 (0.13-2.44) |  | 11 | 6 | 1.88 (0.61-5.79) |

^a^ Adjusted for age at interview, age at menarche, age at first birth, body mass index and first-degree family cancer history, shift work and daytime dietary factors (consumption of cereals, deep-fried foods, preserved meats and dairy products).

* Significant level at *P* value <0.05.
