# Supplementary material for: Nighttime eating and breast cancer among Chinese women in Hong Kong
Source: Breast Cancer Res. 2017 Mar 17;19:31. doi: 10.1186/s13058-017-0821-x (PMC5356318; doi:10.1186/s13058-017-0821-x)
Supplement: Additional file 4: Table S3. — Associations between nighttime eating and breast cancer stratified by ER status. (DOCX 14 kb) [file 13058_2017_821_MOESM4_ESM.docx]

Additional File 4

Table S3 Associations between nighttime eating and breast cancer risk stratified by ER status

| Variables | Controls | ER+ breast cancers | |  | ER- breast cancers | |
| --- | --- | --- | --- | --- | --- | --- |
|  | (N=913) | (N=601) | OR (95% CI)^a^ |  | (N=183) | OR (95% CI)^a^ |
| Nighttime eating after 10pm |  |  |  |  |  |  |
| Never | 774 | 491 | 1.00 (ref) |  | 157 | 1.00 (ref) |
| Ever | 139 | 110 | 1.54 (1.04-2.28)* |  | 26 | 1.13 (0.63-2.03) |
| Meal type |  |  |  |  |  |  |
| Never | 774 | 491 | 1.00 (ref) |  | 157 | 1.00 (ref) |
| Staple food | 86 | 86 | 2.20 (1.37-3.53)* |  | 19 | 1.52 (0.77-3.03) |
| Snacks | 57 | 30 | 0.88 (0.48-1.63) |  | 7 | 0.52 (0.17-1.54) |
| Food type |  |  |  |  |  |  |
| Never | 774 | 491 | 1.00 (ref) |  | 157 | 1.00 (ref) |
| Noodles | 40 | 46 | 2.64 (1.39-5.04)* |  | 12 | 2.02 (0.83-4.84) |
| Rice | 37 | 48 | 2.84 (1.49-5.43)* |  | 7 | 1.53 (0.56-4.18) |
| Baked goods | 37 | 22 | 1.41 (0.73-2.70) |  | 3 | 0.36 (0.08-1.60) |
| Meat | 23 | 26 | 2.05 (0.94-4.49) |  | 3 | 0.96 (0.26-3.63) |
| Vegetable | 22 | 19 | 1.15 (0.77-3.26) |  | 3 | 1.04 (0.20-5.29) |
| Fruit | 13 | 9 | 1.06 (0.38-2.99) |  | 3 | 0.96 (0.20-4.65) |

^a^ *A*djusted for age at interview, age at menarche, age at first birth, body mass index and first-degree family cancer history, shift work and daytime dietary factors (consumption of cereals, deep-fried foods, preserved meats and dairy products).

* Significant level at *P* value <0.05.
